# Uncommon Side Effects of COVID-19 Vaccination in the Pediatric Population

**DOI:** 10.7759/cureus.30276

**Published:** 2022-10-13

**Authors:** Trupti Pandit, Ramesh Pandit, Lokesh Goyal

**Affiliations:** 1 Pediatrics, Nemours Children's Health, Glen Mills, USA; 2 Medicine, Independent Researcher, Philadelphia, USA; 3 Hospital Medicine, University of Pennsylvania / Chester County Hospital, Philadelphia, USA; 4 Hospital Medicine, CHRISTUS Spohn Hospital Corpus Christi - Shoreline, Corpus Christi, USA

**Keywords:** covid-19 vaccine side effects, prevention, mis-c, pediatric, appendicitis, menstrual disorder, seizures, stroke, myocarditis, covid-19 retro

## Abstract

Introduction

The rapid development of vaccines followed the Coronavirus disease 2019 (COVID-19) pandemic. There is still significant vaccine hesitancy, especially among parents. Large-scale pediatric population-based studies or reviews about vaccine side effects are limited.

Data sources and methods

The Centers for Disease Control and Prevention (CDC) recommends recipients or their providers notify possible adverse events to the Vaccine Adverse Event Reporting System (VAERS). We evaluated Delaware state data from the VAERS system for the pediatric age group.

Results

A total of 111 reports were reviewed, with summaries of the reported key side effects discussed, including seizures, myocarditis, stroke, multisystem inflammatory syndrome in children (MIS-C), chest pain, hematuria, menstrual disorder, appendicitis, behavioral and otological side effects, etc.

Conclusions

We noted the approximate prevalence of reported adverse events to be <0.2%. Further studies with larger sample sizes or those focused on each key side effect are needed to evaluate these side effects in detail. An open discussion about the possible side effects and reinforcing the individual, family, and community benefits are key to promoting COVID-19 vaccine acceptance.

## Introduction

The Corona Virus Disease 2019 (COVID-19) pandemic has resulted in widespread health, social, and economic impacts [1]. It also led to the rapid development and emergency use authorizations of the vaccine against coronavirus (SARS-CoV-2) [2]. The vaccines underwent an expedited review process and were released for general use to control the pandemic. The evaluation of the side effect profiles followed proper procedures, but the sample groups, especially in children, were not as extensive as prior vaccines, possibly due to the newer form of vaccines and urgency at the time [3]. There have been isolated studies about vaccines and certain adverse outcomes [4]. Similar analyses were performed for the adult population [5]. Still, a detailed evaluation of population-level reporting in the pediatric age group is lacking. We aim to review the reported side effects for the pediatric age group to inform the clinicians and the general population about possible side effects and provide detailed information for objective decision-making.

## Materials and methods

With the vaccine deployment, the Centers for Disease Control and Prevention (CDC) also created a section for the COVID-19 vaccines in the Vaccine Adverse Event Reporting System (VAERS) [6]. Patients/parents/guardians, and/or healthcare providers reported suspected side effects to the VARES. The VAERS data is openly available for researchers. The decision was made to limit the search scope to the Delaware state and the year 2021 due to resource limitations. Due to the dataset format, the age group was limited to 6-17 years.

Inclusion criteria

We queried the VAERS system with the terms listed below in Table 1.

**Table 1:** VAERS Wonder System Query Terms VAERS: Vaccine Adverse Event Reporting System

Term	Description
Query Date	Apr 04, 2022
Age	6-17 years
Date Report Completed	2021
State/Territory	Delaware
Vaccine Products	COVID-19 VACCINE (COVID-19)
Group By	VAERS ID; Event Category; Age; Sex

Literature search

We performed a literature search in the PubMed index and Google scholar for each side effect under consideration for this article. The Search terms used were ‘COVID-19’ OR ‘SARS-CoV-2’ OR ‘COVID’ AND ‘vaccines or vaccination’ AND ‘adverse Effects’ OR ‘side Effects’ AND ‘myocarditis’, ‘stroke’, ‘seizure’, ‘menstrual disorder’, ‘chest pain’, ‘allergic reaction’, ‘appendicitis’, ‘behavioral’, ‘Multisystem inflammatory syndrome in children (MIS-C)’, AND ‘hematuria’.

We included the following article types: Case reports, case series, cohort studies, and clinical trials. We also reviewed the Food drug administration (FDA), and Centers for Disease Control (CDC) website information. The Institutional Review Board of Inspira Health reviewed and approved the study protocol, File #2022-05-003.

## Results

The final cohort included 111 patients. Life-threatening events such as stroke and seizures were rare, and none of the reports included death. Further details of the individual report's exact age or racial profile were unavailable in the dataset. Although no direct comparison was made with the number of vaccinations, as per the State of Delaware records, a total of 62013 doses had been administered by 12/31/2021 to the age group of 5-17 years [7]. Acknowledging the dataset limitations and the age group discrepancy, the adverse events reported had an approximate incidence of 0.18% or less than two per 1000 vaccine recipients.

Among the reported events, the majority were concerning the vaccine delivery/administration, including the wrong brand of vaccine or the wrong dose of vaccine administered (n=48). Excluding dosing or administration issues, details of the 63 reports were reviewed (Table 2). The key part to note is that given that some cases had reported multiple complaints, the total side effects reported below will be more than the 63 total reports.

**Table 2:** Reported Side Effects in the VAERS Query Results VAERS: Vaccine Adverse Event Reporting System

System/Side effects	Number	System/Side effects	Number
Cardiac		General	
Chest pain	11	Fever	5
Myocarditis	3	Chills	4
Elevated troponin	3	Fatigue	3
Palpitation	3	Lymphadenopathy	3
Hypotension	2	Muscle pain	1
Gastroenterology		Integumentary/Autoimmune	
Nausea	6	Allergic reaction	7
Loss of appetite	2	Swelling at the injection site	1
Elevated Bilirubin	1	Pulmonary	
Appendicitis	1	Shortness of breath	4
Glucose levels Fluctuation with DM -1	1	Wheezing	1
Neurology		Genito-Urinary	
Syncope	9	Dysmenorrhea	5
Dizziness	6	Hematuria	1
Headache	6	Orthopedic & rheumatology	
Seizures / Involuntary movements	4	Arm/shoulder pain	2
Lightheadedness	3	Joint pain	2
Blurry Vision	3	Muscle pain	1
Stroke	1	Otological symptoms	
Psychiatry		Muffled hearing	1
Worsening of existing behavioral disorders	1	Ringing in ear	1

Some of the selected side effects, based on importance or uncommon but serious nature, are discussed in detail below, with a literature review regarding the topic providing further commentary. We created a citation [8] to refer to the resulting dataset for ease of expression.

Multisystem inflammatory syndrome in children (MIS-C)

As per the report received from a healthcare professional, after an unspecified vaccination duration, one patient reported COVID-like symptoms, fever, vomiting, rash, and diarrhea [8]. After a few days, the patient developed respiratory, gastrointestinal, dermatologic, and neurologic symptoms, requiring hospitalization, and was diagnosed with MIS-C.

According to Levy et al. study, a single COVID-19 vaccine injection to MIS-C onset interval was 25 days [9]. However, in Levy et al. study, there are no MIS-C cases in fully vaccinated children. After SARS-CoV-2 infection, a mean 28-day delay was noticed to MIS-C onset [10]. The incidence of MIS-C was one per million in individuals who received one or more doses of the COVID-19 vaccine compared to 200 per million cases of MIS-C in unvaccinated individuals [11].

Appendicitis

A patient received the BNT162b2 vaccine a few days before presenting with an upset stomach and vomiting [8]. The patient was diagnosed with appendicitis and underwent an appendectomy.

Cases of appendicitis have been reported post-COVID-19 vaccination [12,13]. One possible mechanism of appendicitis is the inflammatory enlargement of lymph nodes in the abdomen following the COVID-19 vaccination [14]. Out of 43,448 participants, eight in the vaccine group suffered from appendicitis following COVID-19 vaccination, and four in the placebo group suffered appendicitis [13]. However, these cases of appendicitis showed no direct association with vaccination, as the frequency of appendicitis post-COVID-19 vaccination was not higher than expected within the general population [13].

Hematuria

An adolescent male reported hematuria two times after his BNT162b2 vaccine. During these episodes, he also had mild stingy sensations with urination [8].

A study reported 27 cases of gross hematuria after the COVID-19 vaccine [15]. Seventy percent of those had a prior diagnosis of immunoglobulin A nephropathy. This study suggested no real progression of hematuria to severe kidney dysfunction.

Stroke

One patient was reported to have had a stroke 28 days after the BNT162b2 vaccination [8]. The patient presented with right-sided weakness and paresthesias, acute embolic stroke, and pulmonary embolism. The patient was later found to have a patent foramen ovale. The additional contributing factor was considered to be the use of oral contraceptives.

The COVID-19 infection is associated with hypercoagulability due to the pro-inflammatory impact and effect on the coagulation cascade [16]. Stroke has been reported with the COVID-19 vaccination as well. Kolahchi et al. summarized eight cases of patients having stroke outcomes with the administration of mRNA vaccination, with the youngest being 36 years of age [17]. The possible pathophysiology for stroke was a combination of atherosclerotic disease and pro-inflammatory status [18].

Seizures

Four cases of seizures were reported [8], although the reports do not clarify any prior history of seizures, limiting the causal association.

Case 1: The patient received the vaccine, and the next day, the patient had a headache and a temperature of 100.0 °F. The patient then had one episode of a seizure lasting 30 seconds, described as the face getting pale, jaw stuck open, and clicking noises, with eyes open and staring up. The patient urinated on herself. After the seizure episode, the patient returned to a normal state.

Case 2: The patient had a seizure two weeks after receiving the vaccine. The patient initially experienced minor undefined seizure episodes, eventually worsening to a full tonic-clonic state. The patient required hospitalization and received anti-seizure medications.

Case 3: The patient had vomiting and focal seizures seven days after receiving the vaccine. No further data was available in the report.

Case 4: The patient started having involuntary movements of arms and legs along with eyes rolling behind his head, with episodes lasting 5 to 10 seconds. The time since vaccination was not specified. The patient initially had only a few episodes, and later, the frequency increased to about 35 seizures-like activities daily. The patient had unremarkable MRI and EEG and was treated with Trileptal.

COVID-19 vaccination has been associated with common neurological symptoms such as dizziness, myalgia, paresthesia, and headaches [19]. These were mostly acute and transient. The incidence rate of COVID-19 vaccine-related seizures was reported to be 3.19 seizures per 100,000 persons per year, and the COVID-19 vaccine increased the risk of new-onset seizures by >30-fold when compared to the influenza vaccine [20]. Most seizures typically occur within two days of vaccination, including influenza and COVID-19. Therefore, caution for those with prior history of seizures is advised.

Menstrual Disorders

A total of five reports were noted for patients with alterations in their menstrual periods [8].

Case 1: The patient received her first BNT162b2 vaccine one month after her menarche and her second dose three weeks later. The patient reported her third menses onset was one day before her second BNT162b2 vaccine. She noticed dysfunctional uterine bleeding, dyspnea on exertion, lightheadedness, and pallor. The patient was hospitalized for symptomatic anemia, requiring a blood transfusion.

Case 2: The patient reported not having a menstrual cycle for two months since getting the COVID-19 vaccine. The patient states that she was due before her initial vaccine dose; however, her menstrual period did not arrive. The pregnancy test was negative. The patient tolerated the BNT162b2 vaccine without any other issues. Other reported cases were similar in presentation to those above.

The Edelman et al. study included 3,959 individuals and reported that compared to the unvaccinated population, the menstrual cycle length was noted to have changed by less than one day in women who received both doses of the COVID-19 vaccine [21]. These changes in the menstrual cycle were noted to be transient. The changes in cycle length did not persist over time [22]. Historically, about 75% of adolescents were affected by menstruation disorders years before COVID-19 and its vaccines [23]. Therefore, reports about COVID-19 vaccination and menstruation disorders should be considered cautiously.

Chest Pain

A total of 11 cases reported chest pain [8].

Case 1: The patient complained of stabbing left-sided chest pain two hours after receiving the first BNT162b2 vaccine and had pain and swelling at the injection site. She denied any history of sarcoidosis, lupus, TB exposure, or other autoimmune diseases. The chest pain and fatigue persisted the following week. The echocardiogram, and blood tests, including troponin, were within normal range, except for elevated Creatinine Kinase (CK) levels at 831 U/L. Repeat testing a few days later noted CK levels had normalized to 76 U/L, and the chest X-ray noted mild right middle lobe atelectasis possibly related to possible viral illness and/or underlying inflammatory process. This further clarifies that this patient likely had the symptoms secondary to viral illness and less likely due to the vaccine. Over the next few days, the patient reported improving her chest pain and denied fatigue, palpitations, dizziness, or myalgia.

Case 2: Three weeks after receiving the second BNT162b2 vaccine, the patient developed chest pain and shortness of breath during sports practice, resolving with rest without recurrence. The patient was evaluated in ER and discharged without needing further intervention.

Case 3: The patient with a history of asthma and food allergies reported wheezing and chest pain one hour after receiving the BNT162b2 vaccine. Symptoms resolved without significant intervention.

Similarly, in Case 4, a patient had reported chest pain following the second dose of the COVID-19 vaccine, which resolved by itself. The other cases were with symptoms similar to those above, with workups negative for myocarditis and normal troponin and BNP levels.

Multiple patients have reported chest pains after the COVID-19 vaccinations, with the majority being noncardiac [24]. Costochondritis is an inflammation of the costal cartilage, a benign cause of reproducible sternal chest pain [25]. This condition might resolve spontaneously or require a short treatment with nonsteroidal anti-inflammatory drugs such as ibuprofen. The Costochondritis symptoms due to COVID-19 infection are more severe than COVID-19 vaccine-related costochondritis [25,26].

Myocarditis

Myocarditis was reported in three cases [8]. Cases 1 and 2 initially presented with chest pain, palpitations, and elevated troponins three days after receiving the second BNT162b2 vaccine. Electrocardiogram (EKG) and echocardiogram were normal in both cases.

Case 3: Chest pain, elevated troponin, shortness of breath, and fatigue was noted 17 days after receiving the first BNT162b2 vaccine. All three instances resolved symptoms with oral nonsteroidal anti-inflammatory drugs (NSAIDs).

Immune response against infection or some other trigger that results in inflammation of the heart muscles is known as myocarditis [27]. Patients with myocarditis usually complain of chest pain, shortness of breath, palpitations, and dyspnea on exertion and have lab evidence of myocardial injury. Myocarditis risk factors include young adults, male gender, diabetes, HIV infection, end-stage kidney disease, and chest injury. The COVID-19 vaccine correlates with myocarditis in pediatric and adult populations [28]. Myocarditis and pericarditis are also seen with other vaccines like smallpox [29] and influenza [30,31].

Cases of myocarditis after receiving the COVID-19 vaccine typically occur within one week of vaccination. Most patients' symptoms resolve themselves with rest and pain medications like NSAIDs [25]. These patients should also follow up with a cardiologist before returning to exercise or sports activities [32].

Allergic Reactions

The key cases with reports of allergic reactions are summarized below [8]. Case 1 was an 11-year-old patient with reported acute onset of swelling of hands and feet, redness, and severe itching associated with itchy red skin on the body. Cases 2 and 3 reported sudden onset of itchy hives all over the body after a few days from vaccination. Case 4 reported a local reaction, with raised erythematous itchy, and painful swelling at the injection site and down on the arm for four days. Case 5 reported acute onset of eyelid redness and swelling, congestion, with red spots on the chest and back. Case 6 reported acute onset of difficulty breathing, throat tightness, and tingling in lips, ears, and throat 10 minutes after vaccination. No breathing compromise was noted. Case 7 reported shakes, redness, throat tightness, and itching. Prior history of allergic reactions to vaccines was not reported in any of the cases. All these cases reported improvement in symptoms with antihistamine administration.

Several studies have reported various cutaneous reactions after BNT162b2 and mRNA-1273 (Moderna) vaccines [33,34]. One possible mechanism of post-COVID-19 vaccine-induced skin lesions is an allergic reaction to vaccine components, with mast cell degranulation causing severe rash, angioedema, and anaphylaxis [35]. Similarly, influenza vaccinations in 2009 were noted to have reports of 10.7 hypersensitivity reactions per million vaccine doses distributed [36]. Those with prior history of allergic reactions were noted to have a higher risk of allergies to mRNA vaccines [37].

Behavioral Issues

A 15-year-old female with a history of pediatric acute-onset neuropsychiatric syndrome (PANS), mast cell activation syndrome, postural orthostatic tachycardia syndrome, obsessive-compulsive disorder (OCD), withholding food, and head-banging behaviors after a few hours of receiving the second COVID-19 vaccine, experienced PANS and OCD symptoms and worsening of behavioral symptoms [8]. She was treated with NSAIDs and an antihistamine (Cetirizine), with the resolution of symptoms a few hours later.

The development of psychological issues has been reported after vaccination, with the influenza vaccine [38] and the yellow fever vaccine [39], including depression, anxiety, and psychosis. The pathophysiology of vaccination-induced psychological/behavioral issues is not entirely understood. However, vaccinations like COVID-19, influenza, etc., stimulate pro-inflammatory cytokines, which have been associated with neuropsychiatric symptoms [40,41].

Fluctuation of Blood Sugar Levels

A 16-year-old diabetes type-1 patient reported a fluctuation in blood glucose level the next day after the vaccine [8]. This was addressed with the sick day insulin plan to control her sugar level.

The COVID-19 vaccine stimulates the immune system to a milder degree than the COVID-19 infection itself, causing stress and affecting hormone levels such as adrenaline, growth hormone, and cortisol, leading to hyperglycemia [42]. Type-1 and Type-2 diabetes patients cannot rapidly counteract high glucose levels, leading to higher fluctuations than patients who do not have diabetes [43]. Immune response-mediated cytokine release can affect blood glucose levels, leading to insulin resistance within tissues [44]. Blood sugar fluctuations after COVID-19 vaccines were noted to be transient, lacking any significant glycemic control impact, with the return to baseline a few days after vaccination [45,46].

Otological Symptoms

One patient reported ringing in the ears, lightheadedness, nausea, and spotty vision for a few minutes after the vaccine administration. The patient had a history of similar symptoms after influenza vaccination. Another patient reported muffled hearing, lightheadedness, and nausea, which resolved in a few minutes [8].

In the Wichova et al. study, 30 patients had new or exacerbated otological symptoms after COVID-19 vaccination. Twelve patients in that study received the Pfizer vaccine, and 18 received the Moderna vaccine. Of 30 patients, 83% reported hearing loss, 50% reported tinnitus, 26% reported dizziness, and 16% reported vertigo [47]. As per the systematic review, the pulled estimate of prevalence was 7.6% for hearing loss, 14.8% for tinnitus, and 7.2% for vertigo based on retrospective recall of symptoms [48].

Abnormal Liver Function Test Results

One patient reported an elevated bilirubin level of 2.04 after the first dose of the Pfizer vaccine [8]. Liver enzymes were not elevated. The patient had reported nausea and reduced appetite for a week after vaccination.

Autoimmune hepatitis after vaccination is rare, and most cases improve after steroid treatment [49,50]. It is possible that, in predisposed patients, COVID vaccines may unmask autoimmune diseases [51]. Antibodies against the spike protein were noted to have affinity against transglutaminase 3, transglutaminase 2, anti-extractable nuclear antigen, nuclear antigen, and myelin basic protein [52].

## Discussion

Multiple COVID-19 vaccine-related side effects are reported in the pediatric population in our reports and similarly in the available literature. Those of most common or clinical relevance, such as rare side effects like myocarditis, costochondritis, hematuria, menstrual disorder, stroke, etc., are summarized above, including the discussion about those in contrast to the reviewed literature.

Per the CDC, VAERS received 9,246 adverse event reports in the age group of 12 to 17 years by 06/16/2021 for Pfizer-BioNTech vaccination [53]. Of these, 90.7% of accounts were nonserious, while 9.3% were serious adverse events, including myocarditis (4.3%). Per this Hause et al. report, adolescents reported local (63.4%) and systemic (48.9%) reactions. During the 3/11/21 to 12/19/21 period, for children aged 5-11 years, VAERS received 4,249 reports of adverse events in this age group, 97.6% of which were not serious [54]. The majority of these were local (57.5%). The systemic reactions, including fatigue and headache, were reported in 40.9%.

We postulate the low prevalence reported in our dataset (approximately <0.2%) to be likely secondary to underreporting minor adverse effects, especially after the initial few months of vaccinations, and some side effects became commonly accepted.

We have no intention of repudiating the overwhelming public health benefits of the COVID-19 vaccines. Vaccines play a major role in controlling the spread and impact of infectious diseases. Considering COVID-19 infection and its complications, vaccination in the pediatric population and pregnant women is needed to minimize the ongoing spread and evolution of the COVID-19 pandemic in the pediatric population [55].

Severe allergic reactions are contraindications for the future administration of the said vaccine [56]. Similarly, careful consideration is also required after the occurrence of significant side effects. Hesitancy against vaccines is common in public and among healthcare workers, with post-vaccination side effects being the key barrier [57]. Open discussion about the possible side effects and reinforcing the individual, family, and community benefits are key to promoting acceptance [58]. Addressing the unique barriers to vaccine acceptance based on community dynamics, sociocultural factors, and vaccine criticism while providing well-informed messaging is vital [59, 60]. For example, appropriately framing the side effect with comparative risk labeling describing the risks associated with COVID-19 or other viral infections can improve vaccine acceptance [61].

There is increasing consensus against mandatory vaccination of healthy children for ethical reasons [62]. Our study also supports this because even though very rare, as discussed above, the COVID-19 vaccine has been correlated with serious side effects and hospitalizations in otherwise healthy children. Especially now that the COVID-19 virus is moving from the pandemic to an endemic disease. We recommend thoroughly pre-screening children before the next vaccination or booster dose and allowing the guidelines to be individualized.

Limitations

We acknowledge the following limitations. a) This review summarises the reported adverse events. Given the narrative nature of the data, it should not be considered a causal association. It carries the inherent limitations of voluntarily reported data without validation. b). Reporting bias and lack of uniformity in the provided information. c). Lack of clinical correlation or individual clinical data. d). Available data is focused on acute or short-term adverse events, and long-term side effects cannot be assessed. Most reports have been in the adult population, and direct comparison is not recommended. e). An analysis of a larger population or a prospective study is needed to understand the side effects better.

Despite the limitations above, it is crucial to overview the already reported adverse events to make informed decisions and take preventive measures for those at-risk, such as the history of allergic reactions, diabetes, neurological, or cardiac conditions. There is a need for further focused studies and long-term assessment of the vaccine-related outcomes to provide further evidence to draw any definitive relationship. Patients with similar reported side effects in the past with other medications or identical health conditions need to be advised of additional caution when considering COVID-19 vaccinations.

## Conclusions

Numerous COVID-19 vaccine-related non-life-threatening side effects have been reported in the pediatric population. We noted that these side effects are uncommon, and the approximate prevalence of reported adverse events was <0.2%. Most reported side effects were mild and transient. Those of most clinical relevance, such as rare side effects like myocarditis, costochondritis, seizures, hematuria, menstrual disorder, appendicitis, MIS-C, etc., are summarized above. Further studies are needed to evaluate these side effects in detail. Considering the severity of COVID-19 infection and its complications, vaccination in general and in the pediatric population has massive and astonishing benefits. At the same time, precautions and better screening of those at risk of side effects from the vaccines are required. Especially now that the COVID-19 virus is moving from the pandemic to endemic disease, we recommend reevaluating the mandatory vaccination policy for healthy children. The children should undergo thorough pre-screen before the next vaccination or booster dose and allow the guidelines to be individualized. Preventive measures that identify those at higher risk, minimize possible side effects, and aim at early intervention are crucial for the wider acceptance of vaccines. Open sharing of available information and discussion regarding the vaccine's role in epidemic control will further decrease vaccine hesitancy and increase confidence in the public health system.
